# Re-challenge of Platinum-based Chemotherapy for Platinum-refractory Patients with Recurrent or Metastatic Head and Neck Cancer: Claims Data Analysis in Japan

**DOI:** 10.36469/jheor.2020.12853

**Published:** 2020-05-20

**Authors:** Makoto Tahara, Issei Doi, Tatsunori Murata, Sari Mishina, Shinji Takai, Hirokazu Kaneko

**Affiliations:** 1 National Cancer Center Hospital East, Kashiwa, Japan; 2 Ono Pharmaceutical Co., Ltd., Osaka, Japan; 3 CRECON Medical Assessment Inc., Tokyo, Japan; 4 Bristol-Myers Squibb K.K., Tokyo, Japan

**Keywords:** head and neck cancer, chemotherapy, platinum-refractory, claims data, re-challenge

## Abstract

**Background:**

The role of platinum rechallenge in head and neck cancer (HNC) has not yet been fully evaluated.

**Objectives:**

It is our goal to assess the real-world treatment patterns and usefulness of platinum rechallenge in patients with platinum-refractory recurrent or metastatic HNC receiving platinum rechallenge.

**Methods:**

This is a retrospective study using data from a Japanese hospital claims database stored in electronic hospital information systems. Patients with HNC or undefined histology with an HNC diagnosis using the disease code, between January 1, 2013 and September 30, 2016, were included. Patients diagnosed with other malignancies on or before the initial diagnosis of HNC and those without cancer stage information in the database were excluded from the study.

**Results:**

A total of 43 994 patients were identified from the database as HNC patients. Of those, in patients who had cancer progression within 6 months after platinum-based chemotherapy administered for primary or recurrent disease (N=842), the median treatment duration of platinum rechallenge for platinum refractory patients was only 1 cycle. The second-line treatment continuation rate at 6 months was 20.1% for patients who received platinum rechallenges and 32.8% for those who received non–platinum-based regimens.

**Conclusions:**

The findings from this study of data from routine clinical practice suggest that the benefit of platinum rechallenge in a platinum-refractory setting would be limited.

## BACKGROUND

Approximately 600 000 new cases of head and neck cancer (HNC) are diagnosed annually worldwide.^1^ Cisplatin plays a central role in chemotherapy for current HNC treatment. In the locally advanced setting, chemoradiotherapy concurrently with cisplatin is regarded as the standard treatment for a high number of patients, including those with resectable HNC in whom organ preservation is the goal; those with unresectable HNC; and those with postoperative HNC with a high risk of recurrence.^2^ However, despite treatment for locally advanced HNC, half of the cases still experience recurrence. Previous studies have shown a median survival of ≤6 months in patients with HNC who had disease progression within 6 months of platinum based chemotherapy.^3^–^5^

A longer interval between prior platinum-based therapy and platinum rechallenge has been shown to be associated with an increase in response to platinum rechallenge in patients with ovarian cancer.^6^ Furthermore, in the relapsed epithelial ovarian cancer setting, there is a certain consensus on the definitions of terms used for treatment standardization. For example, “platinum-refractory” is defined as cases in which the disease progresses during platinum-based therapy; “platinum-resistant” is defined as cases in which the disease relapses within 6 months after the end of platinum treatment; and “platinum-sensitive” is defined as cases in which the disease relapses at least 6 months after the end of platinum treatment. However, there is no established definition of “platinum-refractory” in the HNC setting, and the role of platinum rechallenge in platinum-refractory HNC remains to be fully elucidated. Thus far, no prospective study has been performed to evaluate the efficacy of platinum rechallenge in patients with platinum-refractory HNC, which is likely attributable to the ethical concerns of a prospective study design in this setting. Therefore, we aimed to perform a study using a Japanese claims database with 44 000 HNC patients, representative of the nationwide population, to assess the real-world treatment patterns and utility of platinum rechallenge in patients with platinum-refractory recurrent or metastatic HNC (R/M HNC) receiving platinum rechallenge.

## METHODS

### Study Design and Data Source

This is a retrospective study of data from a Medical Data Vision Co., Ltd. (MDV; Tokyo, Japan) claims database. The MDV database is a nationwide hospital-based insurance claims database covering approximately 19 million patients treated as inpatients and outpatients at 300 hospitals in Japan (as of May 2017) participating in the Diagnosis Procedure Combination (DPC) payment system/per-diem payment system (PDPS) in Japan. The MDV database contains an anonymized patient identifier, along with information on patient gender, birth year, department visited, date of medical service, diagnosis code(s), hospitalization, medical procedures and test orders, operations, and prescriptions.^7^

The data extraction period for the analysis was defined as the period after biologic drug (cetuximab) approval for HNC in Japan to minimize the calendar effects due to the change in treatment standards (between January 1, 2013 [after cetuximab approval for HNC] and September 30, 2016 [before nivolumab approval for HNC]).

### Study Population

All patients diagnosed with HNC (International Classification of Diseases, 10th Revision [ICD-10] code C00x for cancer of the lip; C01x–C06x for cancer of the oral cavity; C07x and C08x for cancer of the salivary glands; C09x–C13x for cancer of the pharynx; C30.0 for cancer of the nasal cavity; C30.1 for cancer of the middle ear; C31x for cancer of the paranasal sinuses; and C32x for cancer of the larynx) in the MDV database were identified. Eligible subjects were then screened on the basis of the following inclusion criteria: (1) HNC or undefined histology with an HNC diagnosis in the data extraction period using the Japanese disease code and (2) age ≥18 years at the initial diagnosis of HNC regardless of cancer staging. Patients diagnosed with other malignancies on or before the initial diagnosis of HNC and those without cancer stage information in the database were excluded from the study. Furthermore, in order to identify the study population for the R/M HNC analysis, follow-up was defined as the period from the first record of stage III, stage IV, or recurrent HNC diagnosis. Patients whose stage information was unknown were excluded from the analysis. Similarly, in order to identify HNC patients treated with platinum-based regimens, follow-up was defined as the period from the first record of platinum agent prescription. Patients whose stage information was unknown were also excluded from the analysis.

For the analysis of HNC patients treated with platinum-based regimens, platinum-refractory populations were defined as patients with records of other systemic therapy within 6 months from the last dosing date of the previous platinum agent in any setting, because information on disease progression, such as radiographs, is not available from the claims database.

### Study Measures

The number of patients who had received any surgery for HNC, radiation, or pre-identified HNC systemic chemotherapy within 6 months from the first diagnosis date of HNC was identified. The proportion of patients treated with each particular systemic chemotherapy regimen for first-line and second-line treatment was calculated only among those who did not undergo surgery or radiation within 6 months after the first diagnosis date of HNC to exclude adjuvant, neo-adjuvant, and chemoradiation cases.

The line of systemic chemotherapy was defined by the temporal relationship and sequencing of treatment regimens using the dates of initiation and discontinuation of chemotherapy and/or cetuximab. First-line systemic chemotherapy was defined as all chemotherapy and/or cetuximab administered during the 7 days after initiation of treatment, termed the “dosing period.” Maintenance systemic chemotherapy, which was administered following first-line therapy, was distinct from second-line therapy and was defined as systemic chemotherapy following 4 or more cycles of first-line therapy. Of note, in the EXTREME trial, the median number of cycles of chemotherapy before starting maintenance systemic therapy was 5; however, in order to represent the conditions in actual clinical practice, in the present study, the minimum number of cycles in the induction period was set as 4. Second-line systemic therapy was defined as treatment that had been switched following 4 or more cycles of first-line therapy, with an interval without chemotherapy and/or cetuximab between 2 consecutive cycles of >6 weeks, and initiation of a new line of therapy or treatment following fewer than 4 cycles of first-line therapy and subsequent administration of a new treatment regimen not including any agent from the first-line regimen, regardless of the time since the end of first-line therapy. Discontinuation of a single drug from a combination regimen was not considered a change in the line of therapy.

### Statistical Analysis

Categorical variables are presented as percentages. Continuous variables are reported as mean, standard deviation (SD), median, interquartile range, or minimum/maximum value. SAS for Windows version 9.4 (SAS Institute Inc) was used for data management and analysis. No statistical hypothesis tests for comparisons were performed because the objective of the study was to describe real-world treatment patterns during the data extraction period.

## RESULTS

### Patient Characteristics

**Figure 1:**
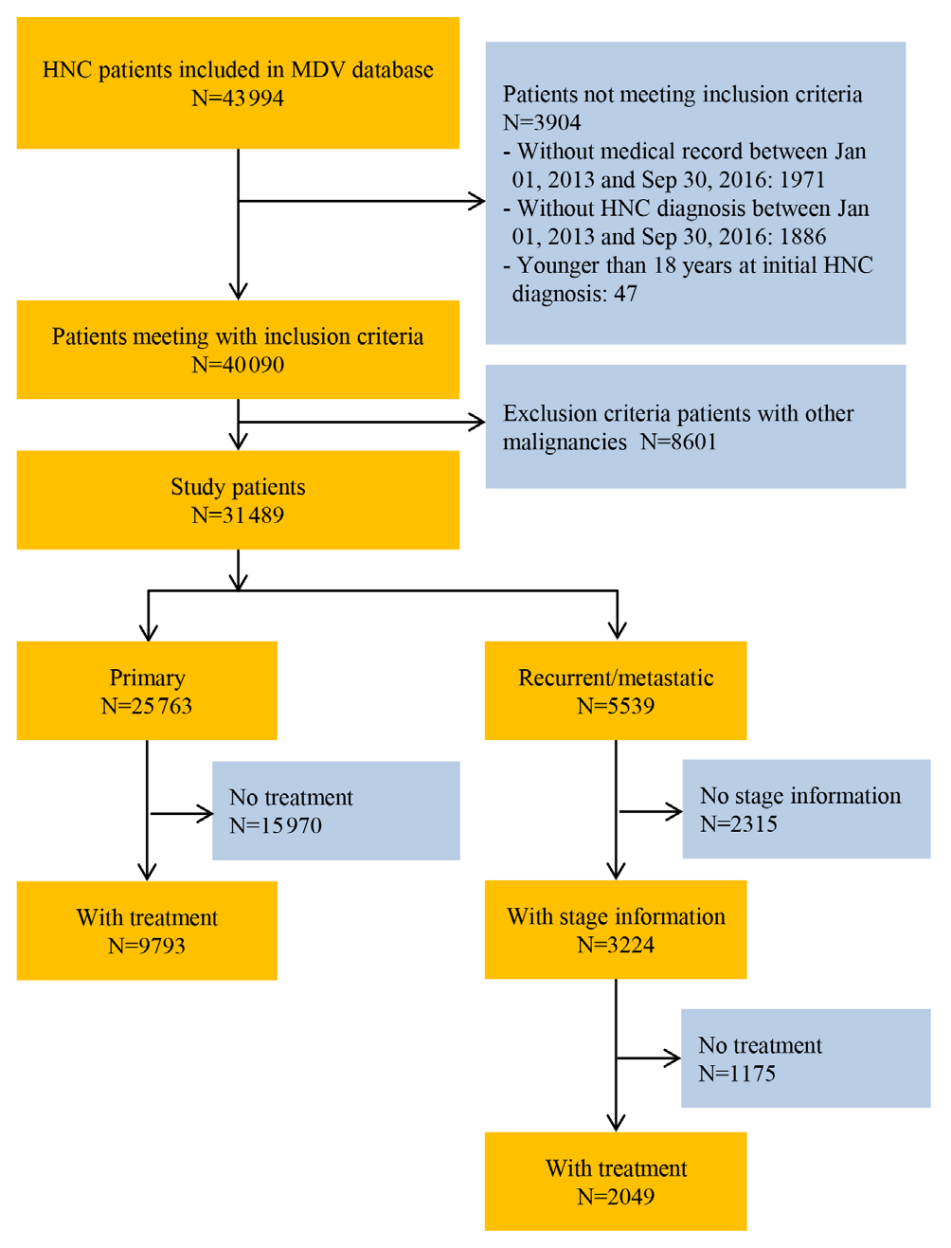
Patient Flow Diagram (Primary and Recurrent or Metastatic HNC) Abbreviations: HNC, head and neck cancer; MDV, Medical Data Vision.

**Figure 2:**
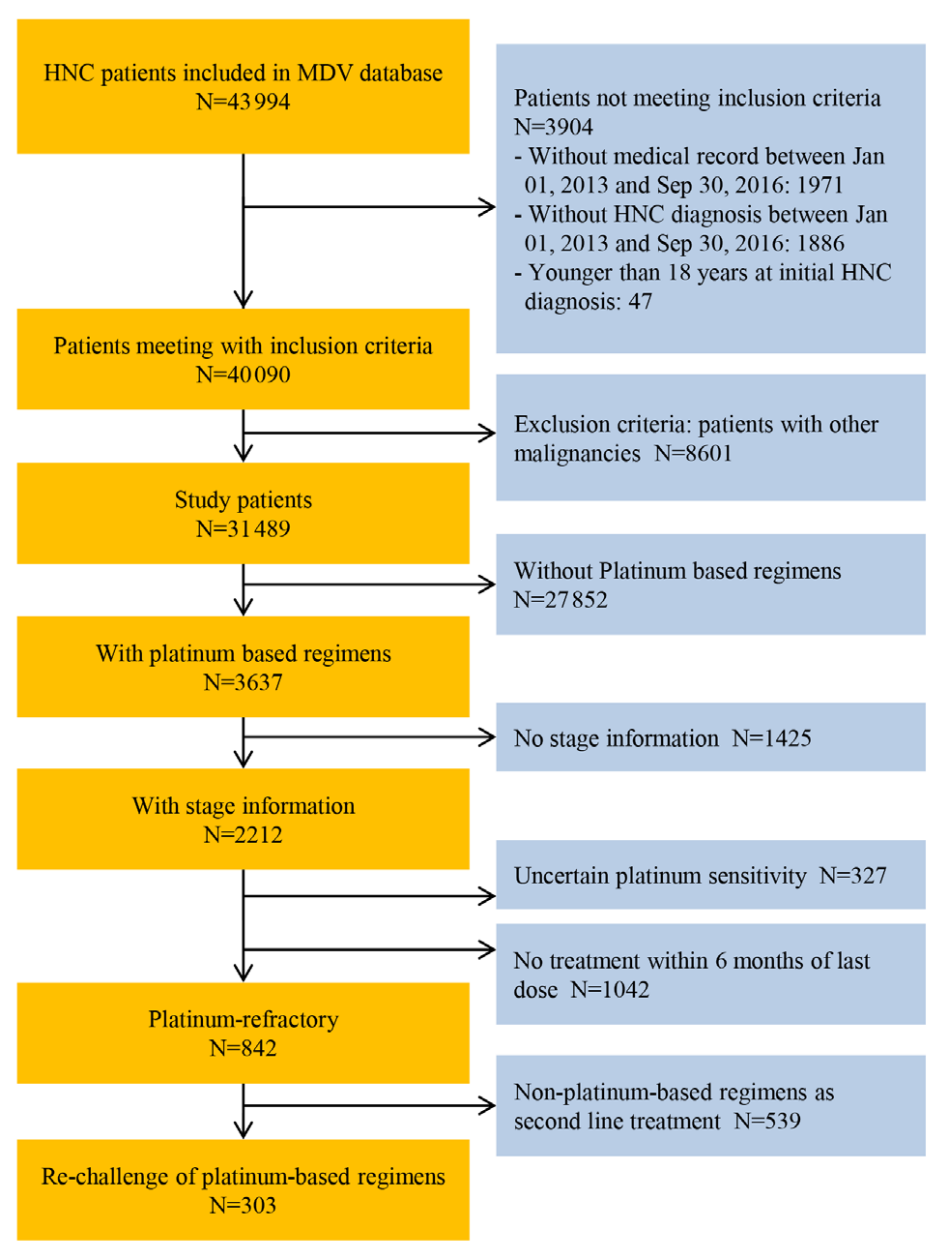
Patient Flow Diagram (Platinum-Refractory HNC) Abbreviations: HNC, head and neck cancer; MDV, Medical Data Vision

A total of 43 994 patients who were diagnosed with HNC between January 1, 2013 and September 30, 2016 were identified from the database. Of these, 8601 (19.6%) patients met the exclusion criteria and were excluded from the analysis. The most frequent malignancies other than HNC were esophageal cancer (n=2575 [29.9%]), gastric cancer (n=1283 [14.9%]), and bronchogenic/lung cancer (n=1152 [13.4%]). Of the 31 489 eligible patients, 3224 were identified as having R/M HNC with stage information and were included in the analysis (Figure 1). A total of 2212 patients with stage information were included in the analysis of HNC patients treated with platinum-based regimens (Figure 2).

Table 1 summarizes the patient characteristics of the R/M HNC patients. In the R/M HNC population (n=3224 patients), the median age was 68.0 years, 80.5% were male, and 70.5% had a history of smoking. According to HNC type, 23.5% of patients had laryngeal cancer, 21.3% had oral cavity cancer, 19.7% had hypopharyngeal cancer, 14.2% had oropharyngeal cancer, 12.6% had maxillary cancer, 9.4% had salivary gland cancer, and 6.4% had nasopharyngeal cancer. Of these patients, 81% were diagnosed with advanced HNC (stage III–IVB) and 15.9% had metastases (stage IVC).

Overall, 38.1% (842/2212) of patients who had stage information were included in the platinum-refractory HNC analysis (Figure 2). The median age of the patients was 65 years, 81.6% were male, and most (76.5%) had a history of smoking. In addition, 20.9% of patients were diagnosed with early-stage HNC (stage ≤II) (Table 1).

### Treatment Patterns in R/M HNC Patients

**Figure 3:**
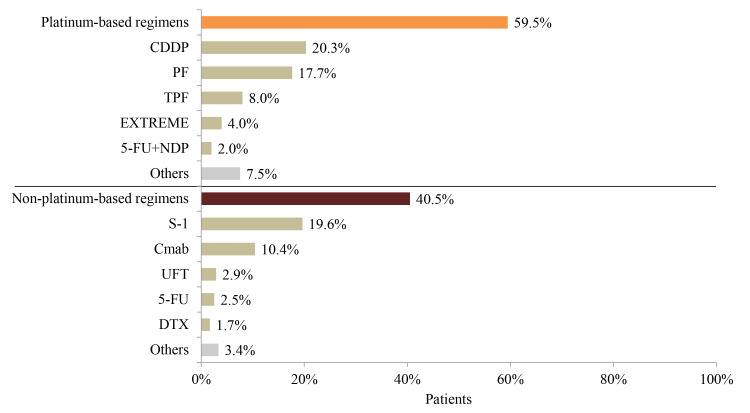
First-Line Treatment Patterns of Systemic Chemotherapy in Recurrent or Metastatic HNC Patients (N=1313) Abbreviations: 5-FU, 5-fluorouracil; CDDP, cisplatin; Cmab, cetuximab; DTX, docetaxel; HNC, head and neck cancer; NDP, nedaplatin; PF, cisplatin+5-FU; TPF, docetaxel+cisplatin+5-FU.

**Figure 4:**
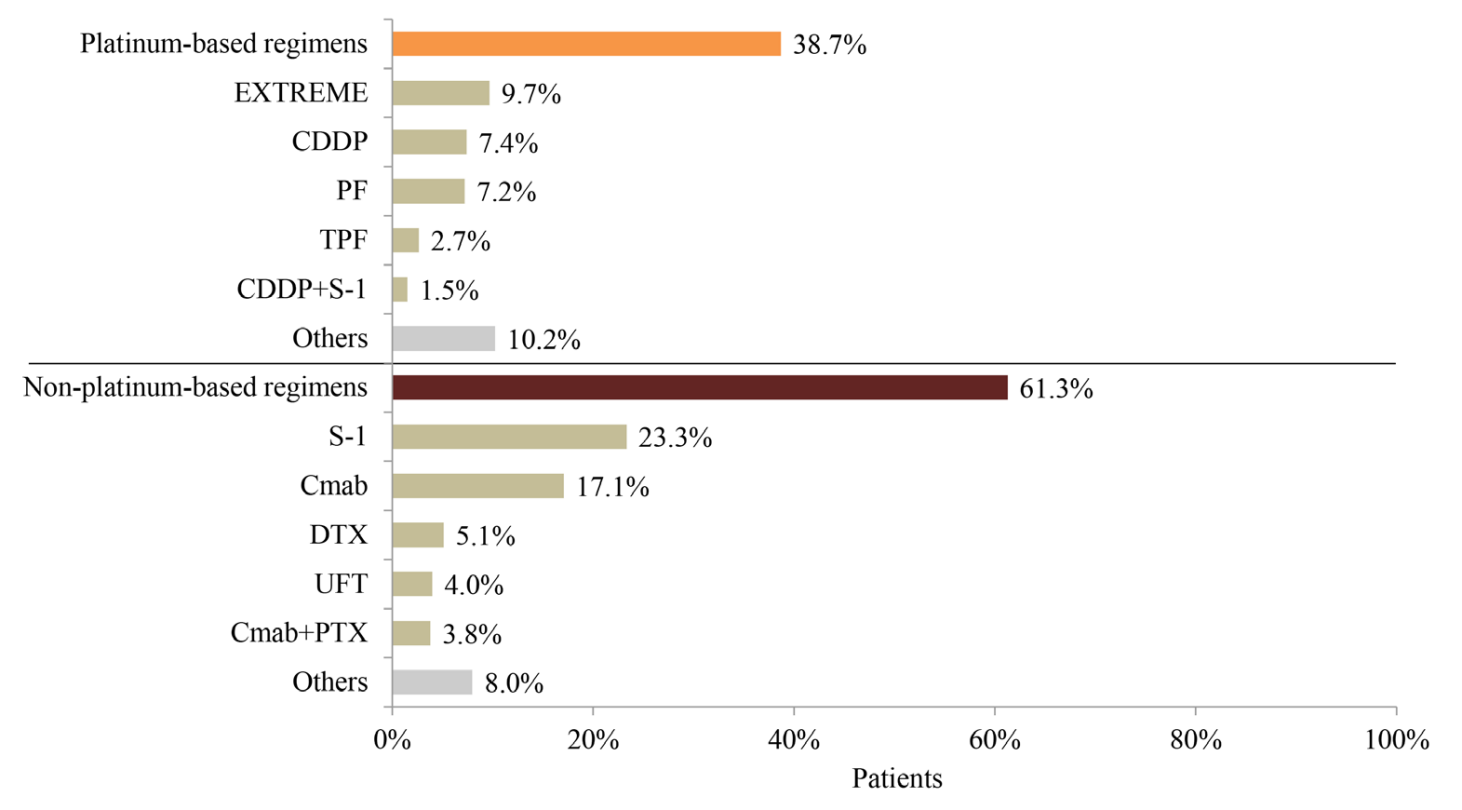
Second-Line Treatment Patterns of Systemic Chemotherapy in Recurrent or Metastatic HNC Patients (N=527) Abbreviations: 5-FU, 5-fluorouracil; CDDP, cisplatin; Cmab, cetuximab; DTX, docetaxel; HNC, head and neck cancer; PF, cisplatin+5-FU; TPF, docetaxel+cisplatin+ 5-FU.

Of the R/M HNC patients who were followed up from the first record of stage III, stage IV, or recurrent HNC diagnosis, 36.4% (1175/3224) did not receive any surgery, radiation, or systemic therapy within 6 months after the first diagnosis of HNC (Figure 1), and 41.6% (852/2049), 56.5% (1158/2049), and 17.3% (355/2049) underwent surgery, radiation, and both, respectively, within 6 months after the first diagnosis. Nearly half (919/2049=44.9%) of the R/M HNC patients received chemotherapy in addition to surgery or radiation, and 19.2% (394/2049) received chemotherapy alone (Table 2). More than 50 chemotherapy regimens were employed. Among the 1313 patients receiving chemotherapy, the majority (59.5%) started with platinum-based regimens, with the most common being cis-diamminedichloroplatinum( II) (CDDP; 20.3%), followed by PF (CDDP, 5-fluorouracil [5-FU]) (17.7%) (Table 2). Approximately 40% of patients received non–platinum-based regimens, with the most common being S-1 (19.6%), followed by cetuximab (10.4%) (Figure 3). Among the second-line treatments for R/M HNC patients, the most common platinum-based and non–platinum-based regimens were EXTREME (9.7%) and S-1 (23.3%), respectively (Figure 4). Only 40.1% (527/1313) of all patients with systemic chemotherapy received second-line therapy. The proportion of non–platinum-based regimens was higher among second-line treatments for HNC compared with first-line treatments (61.3% vs 40.5%).

### Treatment Patterns in Platinum-Refractory HNC Patients

**Figure 5:**
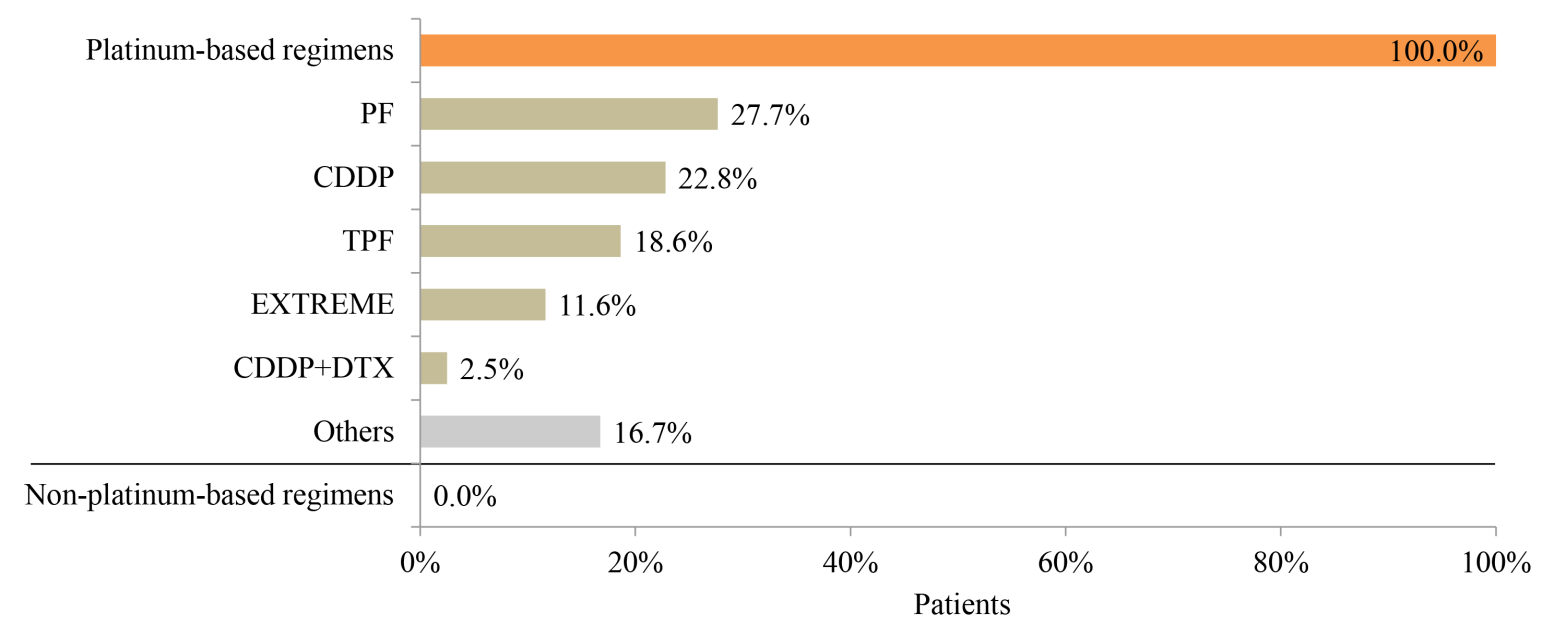
Previous Platinum-Containing Treatment for Platinum-Refractory Patients (N=842) Abbreviations: 5-FU, 5-fluorouracil; CDDP, cisplatin; DTX, docetaxel; PF, cisplatin+5-FU; TPF, docetaxel+cisplatin+5-FU.

**Figure 6:**
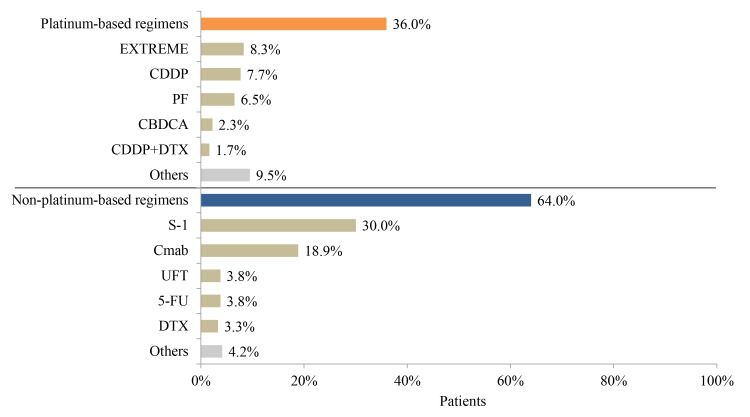
Treatment Regimens for Platinum-Refractory Patients (N=842) Abbreviations: 5-FU, 5-fluorouracil; CBDCA, carboplatin; CDDP, cisplatin; Cmab, cetuximab; DTX, docetaxel; PF, cisplatin+5-FU.

Before becoming refractory to platinum, PF (27.7%), CDDP (22.8%), and TPF (docetaxel [DTX], CDDP, 5-FU) (18.6%) were the most common therapeutic regimens for platinum-refractory patients (Figure 5). Among these patients, 36.0% were re-treated with a platinum-based regimen, including EXTREME, CDDP, PF, carboplatin (CBDCA), or CDDP+DTX, whereas 64.0% received non–platinum-based regimens. The most common non–platinum-based regimen for platinum-refractory patients was S-1 (30.0%) (Figure 6).

**Figure 7:**
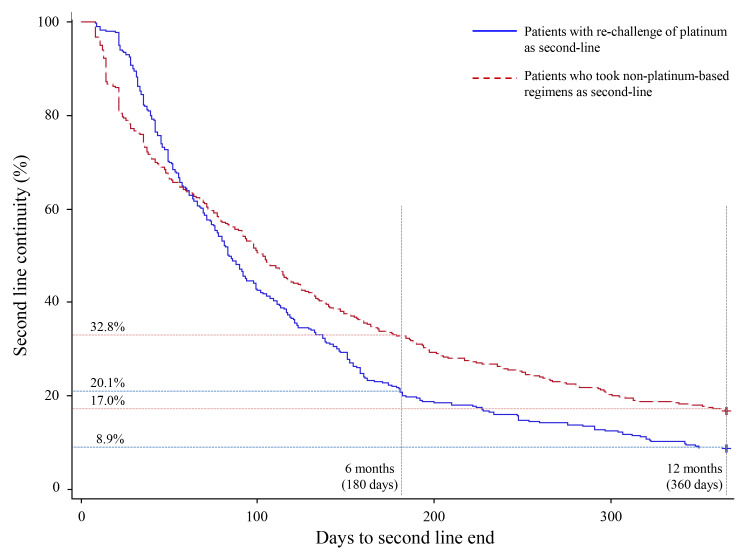
Kaplan–Meier Estimate of Treatment Continuation Rate for Platinum Rechallenge and Treatment Without Platinum

**Figure 8:**
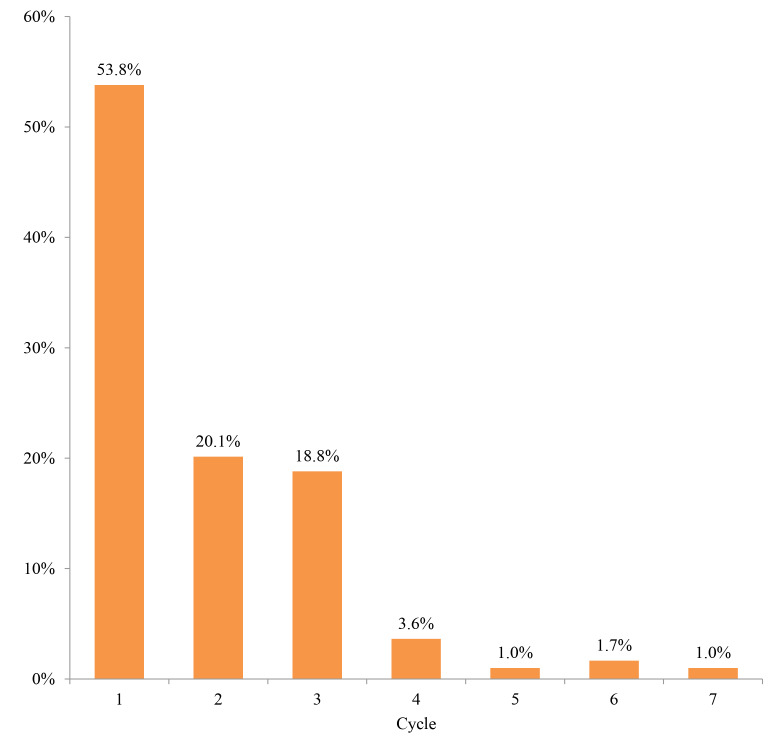
Treatment Cycles of Platinum Rechallenge Treatment Regimens for Platinum-Refractory Patients (N=303)

The second-line treatment continuation rate at 6 months was 20.1% for patients who received platinum rechallenges (including those who proceeded to third-line therapy regardless of whether it was platinum-based) and 32.8% for those who received non–platinum-based regimens (Figure 7). The median number of treatment cycles for platinum rechallenge was one, and the treatment was not continued long-term (Figure 8).

## DISCUSSION

This is the first study to evaluate the real-world treatment patterns of platinum rechallenge in platinum-refractory patients with R/M HNC. No prospective study has been performed to evaluate the efficacy of platinum rechallenge in these patients.

A previous study reported that 10% to 15% of patients had progressive disease within 6 months of first-line platinum-based therapy, with a poor prognosis. Until recently, platinum-refractory patients had limited options other than non–platinum monotherapies.^8^–^11^ However, in the present study, approximately 36% (303/842) of platinum-refractory patients received platinum rechallenge as second-line therapy. One of the reasons could be that the treatment policies varied among institutes and there was no nationwide consensus (1) on the treatment of platinum-refractory HNC during the data extraction period between 2013 and 2016 or (2) on the definition of “platinum-refractory.”

Furthermore, interestingly, the median treatment duration of platinum rechallenge for platinum-refractory patients was only 1 cycle, indicating that platinum rechallenge may not be beneficial for most patients in real-world, probably due to poor clinical activity. Several single-arm studies in platinum-refractory HNC patients have reported that the median treatment duration of non–platinum-based regimens such as weekly paclitaxel, docetaxel, S-1, and cetuximab was approximately 100 days each.^4^,^8^,^12^,^13^ Further consideration of treatment choice in terms of the risk-benefit balance is required for appropriate systemic therapy in platinum-refractory patients.

This study has several limitations, mainly owing to the use of data from a claims database. First, as MDV data used in this study were collected from each contracted hospital in Japan, if patients changed their hospital or visited a clinic/other hospital during the follow-up period, information for that time or for the other hospital was missing. Therefore, there is a possibility of overestimation of the proportion of patients without treatment or a potential bias for generalization. Second, we also confirmed that a duration of ≥4 cycles for platinum-based regimens was rare. As such, misclassification of each regimen as maintenance therapy or second-line therapy is possible (eg, cetuximab as second-line therapy in one regimen may correspond to maintenance therapy in the EXTREME regimen [Figures 4 and 6]). Accordingly, the proportion of patients using other platinum-based regimens or S-1 may have been underestimated. Third, cancer stage information in the MDV database is recorded only on hospitalization and is not required for claims purposes. Patients without stage information were excluded, but the degree of bias in this method is unknown. Furthermore, radiographic information to identify disease progression is not available from the database. Therefore, the results of the present study may not fully reflect the true treatment patterns among platinum-refractory patients (eg, progression during platinum-based treatment as recurrence within 6 months after the last dose of a protocol containing platinum).

## CONCLUSION

Among HNC patients who had disease progression within 6 months after platinum-based chemotherapy administered for primary or recurrent disease, the median treatment duration of platinum rechallenge for platinum-refractory patients was only 1 cycle, indicating that platinum rechallenge would not be beneficial for many patients in real-world settings.

## Figures and Tables

**Table 1:** Characteristics of Recurrent and Metastatic HNC Patients and Platinum-Refractory Patients

		Recurrent and Metastatic HNC Patients		Pt-Refractory Patients	
		Mean	%	Mean	%
Total N		3224		842	-
Gender	Male	2594	80.5%	687	81.6%
	Female	630	19.5%	155	18.4%
Age (year)	Mean	67.5	-	62.7	-
	SD	12.1	-	10.9	-
	Minimum	18.0	-	22.0	-
	Median	68.0	-	65.0	-
	Maximum	99.0	-	90.0	-
History of smoking	Yes	2274	70.5%	644	76.5%
	No	950	29.5%	190	22.6%
Type of cancer	Oral cavity	687	21.3%	178	21.1%
	Maxillary	406	12.6%	107	12.7%
	Oropharynx	458	14.2%	134	15.9%
	Hypopharynx	635	19.7%	234	27.8%
	Larynx	759	23.5%	165	19.6%
	Salivary gland	302	9.4%	45	5.3%
	Nasopharynx	207	6.4%	93	11.0%
Stage^a^	0	2	0.1%	2	0.2%
	I	47	1.5%	54	6.4%
	II	48	1.5%	120	14.3%
	III	1266	39.3%	194	23.0%
	IVA	975	30.2%	197	23.4%
	IVB	372	11.5%	115	13.7%
	IVC	514	15.9%	160	19.0%

Abbreviations: HNC, head and neck cancer; Pt, platinum; N, number; SD, standard deviation.

a The cancer stage number refers to each patient’s first diagnosis.

**Table 2:** Proportion of R/M HNC Patients with Surgery, Radiation, or Chemotherapy Within 6 Months After Dirst Diagnosis of HNC

	N	%
Total	2049	-
Non-drug treatment		
Surgery	852	41.6%
Radiation	1158	56.5%
Surgery + radiation	355	17.3%
Chemotherapy treatment		
Chemotherapy	1313	64.1%
Chemotherapy alone	394	19.2%
Surgery or radiation or both + chemotherapy	919	44.9%

Abbreviations: HNC, head and neck cancer; N, number; R/M, recurrent or metastatic.
